# Protein expression of ABCC2 and SLC22A3 associates with prognosis of pancreatic adenocarcinoma

**DOI:** 10.1038/s41598-019-56059-w

**Published:** 2019-12-24

**Authors:** Lenka Cervenkova, Ondrej Vycital, Jan Bruha, Jachym Rosendorf, Richard Palek, Vaclav Liska, Ondrej Daum, Beatrice Mohelnikova-Duchonova, Pavel Soucek

**Affiliations:** 1 Biomedical Centre, Faculty of Medicine in Pilsen, Charles University, Pilsen, Czech Republic; 2 Department of Pathology, Third Faculty of Medicine, Charles University, Prague, Czech Republic; 3 Deparment of Surgery, Faculty Hospital and Faculty of Medicine in Pilsen, Charles University, Pilsen, Czech Republic; 4 Department of Pathology, Faculty Hospital and Faculty of Medicine in Pilsen, Charles University, Pilsen, Czech Republic; 5 Department of Oncology and Institute of Molecular and Translational Medicine, Faculty of Medicine and Dentistry, Palacky University, Olomouc, Czech Republic

**Keywords:** Tumour biomarkers, Prognostic markers

## Abstract

ATP-binding cassette (ABC) and solute carrier (SLC) transporters translocate diverse substances across cellular membranes and their deregulation may cause drug resistance of cancers. This study investigated significance of protein expression and cellular localization of the previously suggested putative prognostic markers ABCC2 and SLC22A3 in pancreatic cancer patients. Protein localization and brush border staining intensity of ABCC2 and SLC22A3 was assessed in tumor tissue blocks of 65 pancreatic cancer patients and associated with clinical data and survival of patients with regard to therapy. Negative SLC22A3 brush border staining in pancreatic tumors significantly increased the risk of both disease progression and patient´s death in univariate analyses. Multivariate analyses confirmed the association of SLC22A3 expression with progression-free survival of patients. A subgroup analysis of patients treated with regimens based on nucleoside analogs suggested that patients with negative brush border staining or apical localization of SLC22A3 in tumor cells have worse overall survival. The combination of positive ABCC2 and negative SLC22A3 brush border staining predicted worst overall survival and patients with positive brush border staining of both proteins had best overall and progression-free survival. The present study shows for the first time that the protein presence and to some extent also localization of SLC22A3 significantly associate with prognosis of pancreatic cancer in both unstratified and chemotherapy-treated patients. The combination of ABCC2 and SLC22A3 brush border staining also needs further attention in this regard.

## Introduction

Pancreatic ductal adenocarcinoma (PDAC, OMIM: 260350) incidence ranks 12^th^ among cancer diagnoses worldwide, but its mortality is predicted to become the second leading cause of cancer death in the USA within the next decade^1,2^. PDAC clinically manifests by late diagnosis, poor prognosis and overall lack of long term response to the systemic chemotherapy.

Overexpression of ATP-binding cassette (ABC) transporters and downregulation of solute carrier (SLC) transporters is suspected to influence effective intracellular concentration of anticancer chemotherapeutics including some targeted agents^3,4^. There are numerous examples of such effects demonstrated in various *in vitro* model systems^5,6^. The resulting chemoresistance of tumor cells leads to progression of the disease to more advanced stages. Thus, discovery of prognostic factors indicating a high risk of such scenarios and development of tools decreasing the chance of chemoresistance or re-sensitizing chemoresistant cells are urgently needed.

In previous studies, we have found dysregulation of transcript levels of several ABC and SLC transporters in tumor tissues of PDAC patients compared to paired adjacent non-tumorous control tissues. Moreover, intratumoral levels of a number of transporters significantly associated with clinical characteristics of patients^7,8^. Most interestingly, combination of high transcript level of ABCC2 (OMIM: 601107) with low SLC22A3 (OMIM: 604842) level significantly predicted worse overall survival (OS) of patients^9^.

Main goal of the present study was to validate on the protein level the previously suggested putative prognostic role of this combination in independent series of PDAC patients. Thus, we determined protein content of these biomarkers in a larger cohort of PDAC patients and compared it with the patient´ survival in order to substantiate further mechanistic studies behind this association.

## Materials and Methods

### Patients

In total, 65 surgically treated patients with histologically confirmed diagnosis of PDAC and available clinical follow up were included into the study. All patients were recruited and underwent surgery and oncological treatment at the Department of Surgery and Oncology, Teaching Hospital and Medical School in Pilsen, between years 2002 and 2016.

The following data on patients were retrieved from medical records: age, sex, date of diagnosis, date of surgery, resection margins, tumor size (pT), lymph node metastasis (pN), distant metastasis (cM), clinical stage, histological grade, adjuvant treatment regimen, and survival. Clinical characteristics of patients are described in Table 1. None of the patients had received neoadjuvant chemotherapy. The progression-free survival (PFS) served as a measure of the treatment outcome. The PFS was defined as the time elapsed between surgical resection and disease recurrence or death. The OS was defined as the time elapsed between surgical resection and death of any cause.

**Table 1:** Clinical data of patients included in this study.

Characteristics	N = 65
Age (median ± S.D.)	66 ± 8
**Gender**	
Female	32
Male	33
**UICC stage**	
Stage IA	4
Stage IB	14
Stage IIA	8
Stage IIB	32
Stage III	6
Unknown	1
**Tumor size (pT)**	
pT1	10
pT2	27
pT3	23
pT4	4
pTx	1
**Lymph node metastasis (pN)**	
pN0	28
pN1-2	28
pNx	9
**Distant metastasis (cM)**	
cM0	60
cMx	5
Grade	
G1	28
G2	29
G3	4
Gx	4
**Resection margins (R)**	
R0	54
R1	11
Chemotherapy	
None	27
Adjuvant	27
Unknown	11

All procedures performed in this study were in accordance with the ethical standards of the Ethical Commission of the Medical Faculty and Teaching Hospital in Pilsen, Czech Republic (approval reference no. 301/2019) and with the 1964 Helsinki declaration and its later amendments or comparable ethical standards. Experimental protocol of this study was also approved by the Ethical Commission of the Medical Faculty and Teaching Hospital in Pilsen, Czech Republic (approval reference no. 301/2019). Informed consent was obtained from all individual participants included in the study.

### Immunohistochemical analysis

Tissue for light microscopy was fixed in 4% formaldehyde and embedded in paraffin using routine procedures. Five micrometer-thick sections were cut from the tissue blocks and stained with hematoxylin and eosin. For the immunohistochemical (IHC) study samples were first processed by Heat Induced Epitope Retrieval (HIER) using Novocastra™ Epitope Retrieval Solutions pH 6 for ABCC2 and pH 9 for SLC22A3 (Leica Biosystems Inc., Buffalo Grove, IL, USA). Then, endogenous peroxidase activity was blocked using Dako REAL Peroxidase-Blocking Solution (Agilent Technologies, Inc., Santa Clara, CA, USA), 5% normal goat serum was used for background blocking (slides for SLC22A3) and slides were incubated with the following primary antibodies: ABCC2 (dilution 1:100, cat. no. ab3373, Abcam, Cambridge, UK) and SLC22A3 (1:100, cat. no. ab124826, Abcam). The polymerized reporter enzyme staining system (Universal Immuno-peroxidase Polymer) with Anti-Mouse and Rabbit N-Histofine® Simple Stain MAX PO (MULTI) (Cosmo Bio USA, Carlsbad, CA, USA) were used for visualization. Slides were counterstained by Gill’s hematoxylin. Appropriate positive and negative controls were employed. First, predominant localization was evaluated as basal or apical. Then staining in brush border was evaluated as negative or positive. Ambiguous samples were excluded from further statistical analysis (n = 5).

### Data analysis

All statistical analyses were performed using SPSS v16.0 Software (SPSS Inc., Chicago, IL). Differences between groups of patients stratified by clinical data were assessed by the ANOVA and the Pearson tests. Survival function was plotted by the Kaplan-Meier method and the Log Rank and Breslow tests were used for evaluation of the compared groups of patients. Multivariate analysis adjusted to clinical factors which significantly modified survival of patients, i.e. resection margins and lymph node metastasis was performed by the Cox regression.

All *P*-values were calculated from two-sided tests. *P*-values lower than 0.05 were considered statistically significant.

## Results

**Figure 1:**
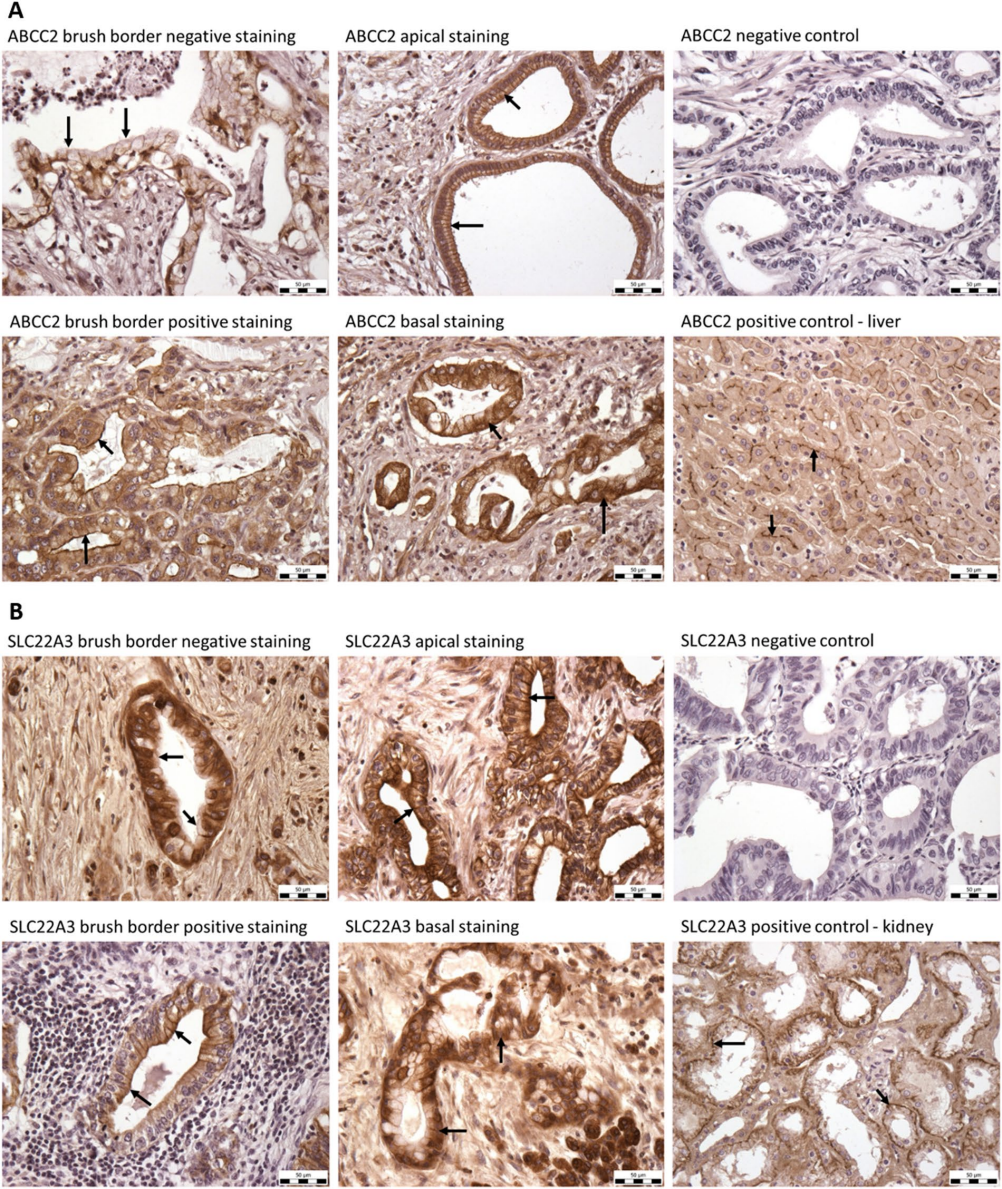
Immunohistochemistry of the ABCC2 and SLC22A3 transporters. (**A**) Examples of ABCC2 negative and positive brush border staining, apical and basal staining, and negative and positive controls. Negative control: tumor tissue stained without primary antibody. Positive control: human kidney cortex. (**B**) Examples of SLC22A3 negative and positive brush border IHC staining, apical and basal staining, and positive and negative controls. Negative control: tumor tissue stained without primary antibody. Positive control: human liver. Arrows allow easier visual control of results. Scale bar 50 µm.

ABCC2 and SLC22A3 proteins were stained by IHC in formalin-fixed paraffin-embedded sections of 65 PDAC patients (clinical data in Table 1). The representative IHC slides are presented on Fig. 1. ABCC2 and SLC22A3 were predominantly localized in basal side of plasma membrane in 49% and 45% samples, respectively. Positive IHC brush border staining was observed in 45% and 29% of patients for ABCC2 and SLC22A3, respectively. Both followed characteristics were then combined.

No significant associations of ABCC2 and SLC22A3 IHC brush border staining or localization with gender and age of the patients, stage, tumor size, presence of local and distant metastasis, tumor grade and resection margins have been observed. The median PFS and OS of all patients were 14 ± 7 and 28 ± 9 months, respectively. Five patients were lost to follow up and were not further evaluated. Patients with R1 resection had significantly worse PFS (*P* = 0.038, Log Rank) and OS (*P* = 0.001) than completely resected (R0) ones. Patients with regional lymph nodes invaded by tumor cells (pN1-2) had significantly worse PFS (*P* = 0.039, Log Rank), but not OS than patients without such invasion (pN0). Therefore, subsequent multivariate analyses were adjusted to lymph node metastasis and resection margins.

**Figure 2:**
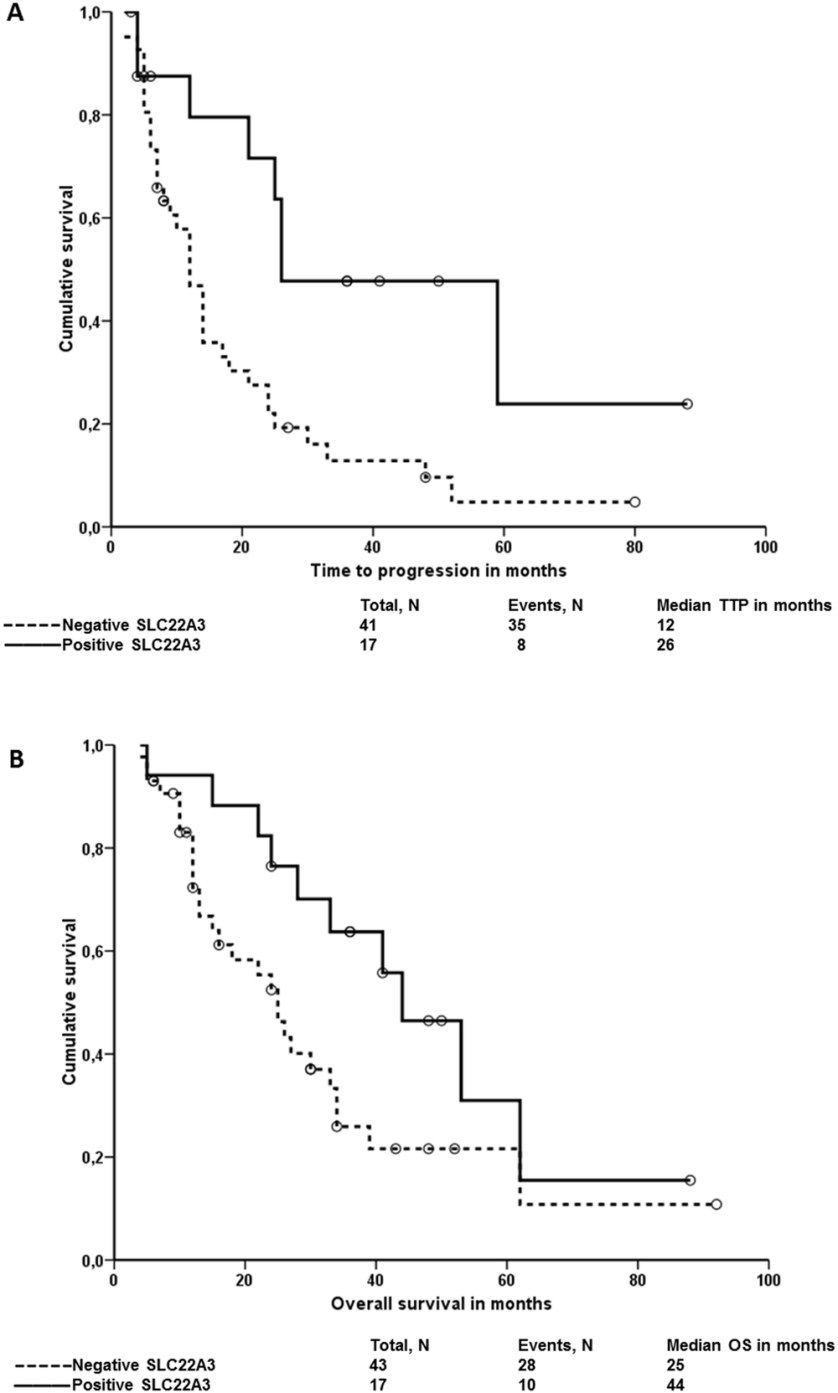
Associations between SLC22A3 brush border staining and the survival of all patients. Kaplan-Meier survival curves were plotted for the PFS or the OS of patients divided into negative and positive staining groups. Dashed line represents the group with negative staining and solid line the group with positive staining. Plot (**A**) shows results of PFS analysis and plot (**B**) of OS analysis.

We then performed univariate analysis of survival functions in groups of patients divided by localization or IHC brush border staining. PDAC patients with negative IHC brush border staining of SLC22A3 had significantly shorter PFS (*P* = 0.041, Log Rank and *P* = 0.024, Breslow; Fig. 2A) and OS (*P* = 0.007, Log Rank and *P* = 0.020, Breslow; Fig. 2B) than patients with positive staining in tumor cells. ABCC2 brush border staining or localization and SLC22A3 localization did not associate with survival of the patients.

Multivariate analysis adjusted to resection margins and lymph node metastasis confirmed that the negative SLC22A3 IHC brush border staining presents high risk factor for disease progression (PFS) (*P* = 0.049, hazard ratio = 2.3, 95% confidence interval = 1.0–5.1), but not for death (OS).

**Figure 3:**
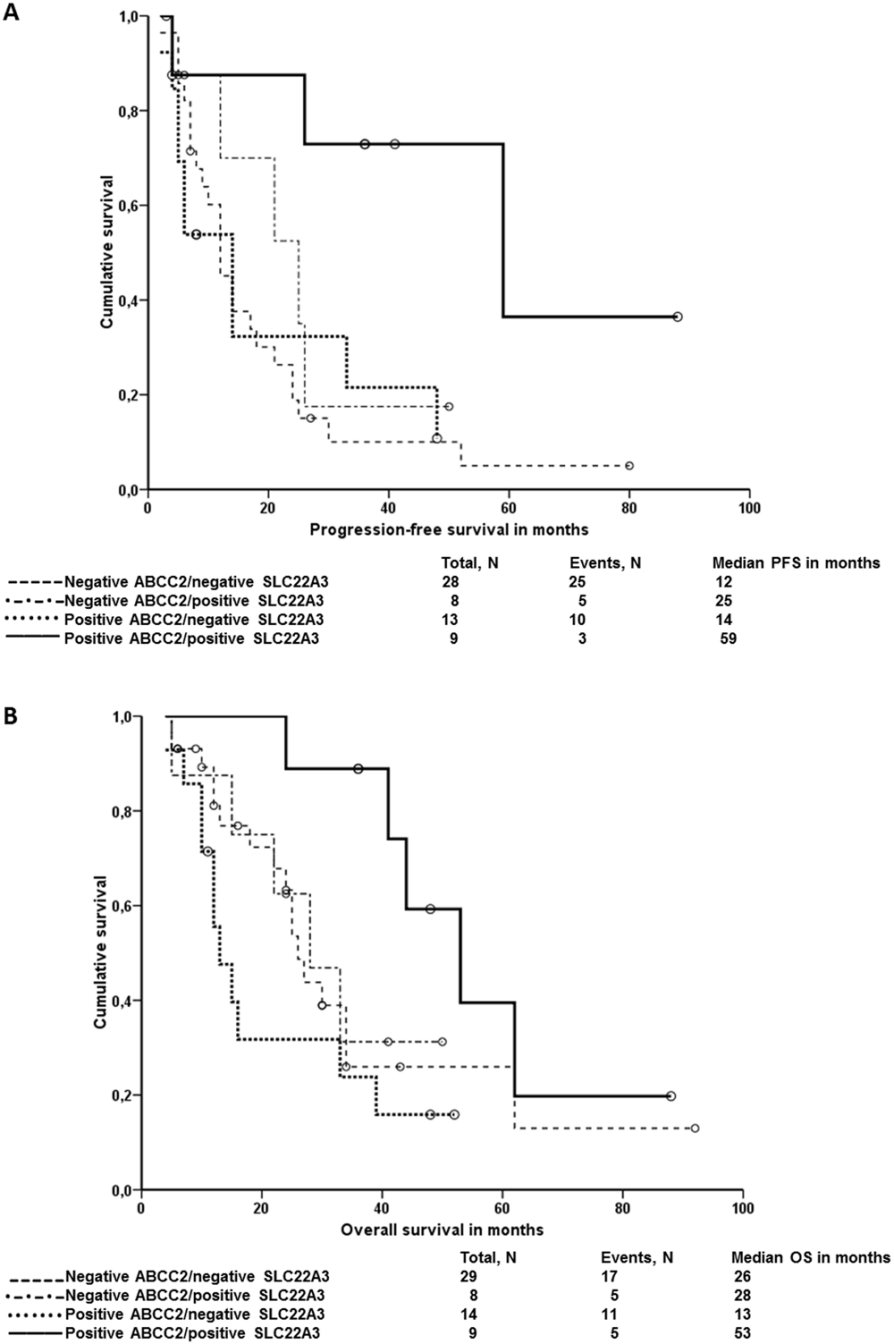
Survival analysis of combinations of SLC22A3 and ABCC2 brush border staining in all patients. Kaplan-Meier survival curves were plotted for the PFS or the OS of patients divided into ABCC2-negative/SLC22A3-negative (dashed lines), ABCC2-negative/SLC22A3-positive (hatched lines), ABCC2-positive/SLC22A3-negative (dotted lines), and ABCC2-positive/SLC22A3-positive (solid lines). Plot (**A**) shows results of PFS analysis and plot (**B**) of OS analysis.

The analysis of combinations of IHC characteristics of both proteins has shown that patients with positive ABCC2 and SLC22A3 brush border staining had the best PFS, but the effect of other combinations was equal (*P* = 0.035, Log Rank and *P* = 0.092, Breslow; Fig. 3A). The combination of positive ABCC2 and SLC22A3 brush border staining was also predictive of best OS, but additionally, patients with combination of positive ABCC2 and negative SLC22A3 intratumoral brush border staining had apparently the worst OS (*P* = 0.048, Log Rank and *P* = 0.013, Breslow; Fig. 3B). The combination of localizations did not influence OS as well as PFS. Multivariate analysis was not performed due to too small numbers of observations in compared subgroups of patients.

**Figure 4:**
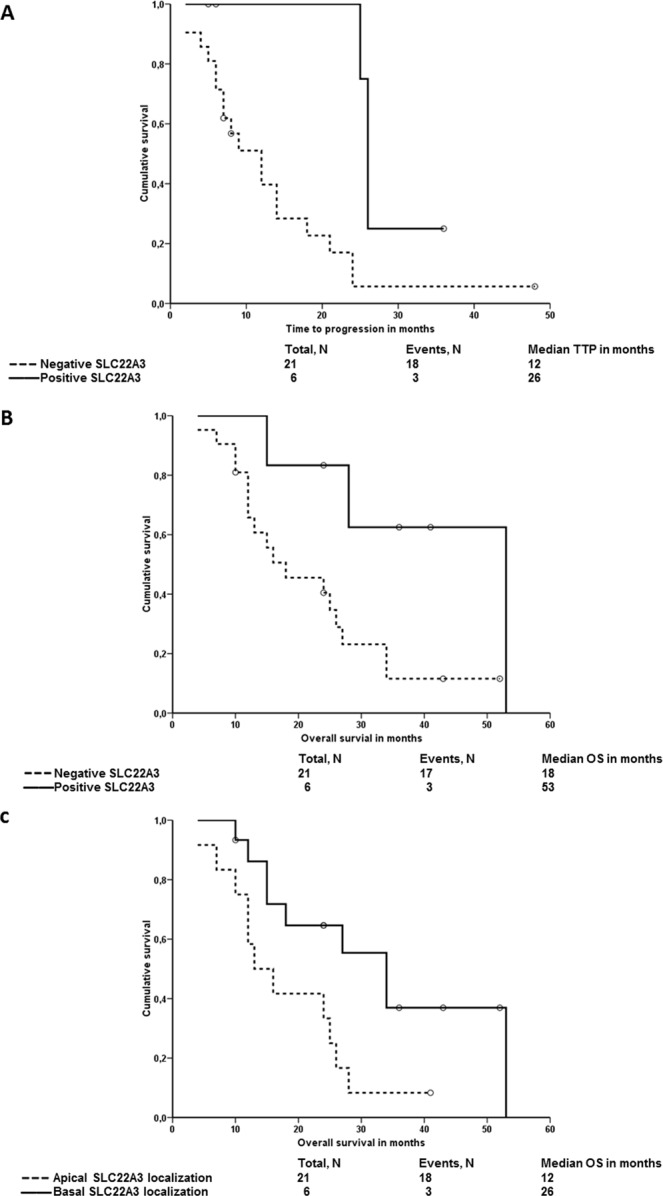
Associations of SLC22A3 brush border staining and localization with the survival of chemotherapy-treated patients. Kaplan-Meier survival curves were plotted for the PFS or the OS of patients divided into negative and positive or apical and basal localization groups. Dashed line represents the group with negative staining, solid line the group with positive staining. Plot A shows results of PFS analysis and plot (**B**) of OS analysis. In the plot (**C**), the solid line represents the group with basal localization and dashed line the group with apical localization of SLC22A3 protein in plasma membrane compared with OS of patients.

Stratified analyses of patients treated with regimens based on nucleoside analogs (gemcitabine and 5-fluorouracil, N = 27) again demonstrated that patients with negative IHC brush border staining of SLC22A3 have significantly worse PFS (*P* = 0.010, Log Rank and *P* = 0.013, Breslow; Fig. 4A) and OS (*P* = 0.030, Log Rank and *P* = 0.040, Breslow; Fig. 4B). Moreover, patients with apical SLC22A3 localization had significantly worse OS than those with basal localization (*P* = 0.021, Log Rank and *P* = 0.029, Breslow; Fig. 4C). Analyses of combinations and multivariate analyses were not performed due to too small numbers of observations in compared subgroups of patients.

## Discussion

Based on the previously observed associations of transcript levels of two less frequently studied membrane transporters with survival of PDAC patients^7–9^, we analyzed prognostic significance of their protein staining intensity and cellular localization by IHC in a larger study. Our data clearly show that presence of SLC22A3 protein associates with prognosis of PDAC patients.

Role of protein expression of SLC22A3 in pancreatic cancer was not addressed to date. Present study demonstrates that patients with detectable SLC22A3 brush border IHC staining in their tumor cells have significantly longer OS and PFS than those without it. SLC22A3 is expressed across a broad spectrum of human tissues mostly in cellular plasma membrane where it actively uptakes a high number of both endogenous substrates and xenobiotics including antineoplastic drugs. Gene expression of SLC22A3 is both genetically and epigenetically regulated^10^. The current view about role of SLC22A3 in cancer has two aspects.

First, SLC22A3 expression was previously connected with onset and progression of several cancers. Specifically, genetic variability of *SLC22A3* predisposes to colorectal and prostate cancer^11–13^. Association of genetic variant rs2504938 in *SLC22A3* with overall survival of pancreatic cancer patients was recently published^14^. Hepatocellular carcinomas express lower SLC22A3 protein levels than non-tumor tissues^15^ and larger size and number of chemically-induced liver tumors were observed in SLC22A3 knock-out mice model compared to wild type mice^16^. In line with these observations, SLC22A3 was reported as metastasis suppressor in familial esophageal squamous cell carcinoma where it directly inhibits ACTN4 (alpha actin 4, OMIM: 604638)^17^. More recently, a correlation between *SLC22A3* promoter hypermethylation and a higher risk for developing familial esophageal squamous cell carcinoma in Chinese was described, together with a new function of SLC22A3 in heat stress-induced oxidative DNA damage^18^. On the other hand, SLC22A3 overexpression promoted cell proliferation and stimulated migration and invasion of colorectal carcinoma cell line models, while repression of the expression reversed these effects^13^. Taken together previous studies suggested that genetic, epigenetic or phenotypic nature of SLC22A3 can serve as putative risk predictive or prognostic biomarker in cancer.

Second, SLC22A3 function as uptake transporter of number of chemicals including antineoplastic drugs was implicated in cancer treatment efficacy and individualization. Higher pre-treatment intratumoral SLC22A3 protein expression was observed in colorectal cancer patients responding to adjuvant chemotherapy with FOLFOX (combination of 5-fluorouracil, folinic acid and oxaliplatin) regimen than in non-responders^19^ suggesting a potential predictive character of this biomarker. In agreement with our findings, patients with head and neck squamous cell carcinoma classified as having high SLC22A3 protein level had improved overall survival after cisplatin therapy and correspondingly, the *in vitro* sensitivity of cell line models to this compound correlated with SLC22A3 expression^20^. Besides frequently used platinum compounds, SLC22A3 also plays a role in the therapeutic action of metformin^21^. High SLC22A3 expression is being tested as a potential surrogate biomarker predicting response of several cancers to metformin^22,23^.

Collectively, the present study and previous reports suggest that SLC22A3 may serve as a putative prognostic and predictive biomarker for follow up validation and functional studies aimed at personalized therapy of PDAC and several other, mostly gastrointestinal, cancers. IHC is routine method established in all pathology departments of larger medical centers worldwide and thus quick application of verified biomarker(s) is envisaged.

Recent genetic study reported that several polymorphisms in the *ABCC2* gene (rs3740067, rs3740073 and rs717620) significantly associate with OS of PDAC patients in an early disease stage and may become prognostic biomarkers^24^. This observation complements previous studies suggesting that *ABCC2* polymorphism rs2273697 associates with poor OS and response to chemoradiotherapy of PDAC patients^25^ and upregulated ABCC2 protein expression leads to an altered sensitivity to gemcitabine and irinotecan *in vitro*^26^. Another study recently pointed out that silencing ABCC2 protein expression increases oxaliplatin accumulation and cytotoxicity in PANC-1 pancreatic cell line model *in vitro*^27^. Although the relation between ABCC2 and PDAC therapy response and prognosis was already well demonstrated in various experimental settings, the present study did not prove association of ABCC2 protein expression alone with survival of PDAC patients.

Prognostic potential of ABCC2 and SLC22A3 combination detected in the present study represents completely new area. Combinations of efflux and uptake transporters by definition of their major functions, i.e. export of drugs outside cells or cellar compartments by ABCs and import of drugs inside by SLCs may have much stronger effect than single biomarker-based predictions. Thus we hypothesized that negative ABCC2 and positive SLC22A3 protein expression should associate with long survival and opposite combination with poor survival of patients. In fact, combination of positive ABCC2 and positive SLC22A3 predicted longest survival (both PFS and OS) suggesting that the extent of overall protein expression dysregulation is prognostic in this case. On the other hand, combination of positive ABCC2 and negative SLC22A3 protein expression indeed predicted shortest overall survival of patients and complied with the function-based hypothesis. However, the breaking up the cohort to very small subgroups poses a problem with statistical power especially in rarely studied diseases and thus, these observations must be replicated.

We are aware of some limitations of our study. First, the modest sample size may be seen as rather low. However, the incidence of PDAC, although quickly rising, is still quite low and mainly the percentage of its resectability is very limited (10–15%). Thus, it is rather difficult to put together larger series of patients with available clinical follow up. The rate of drop-out of patients from the follow up due to very dismal character of the disease is also quite high. Second, numerous systems of evaluation of protein appearance in tumor cells were published and especially in experimental and less studied proteins it is complicated to decide which of the reported approaches should be used. Therefore, we have chosen both semi-quantitative and qualitative measures to robustly assess relevance of proteins of interest. It is necessary to conduct next studies to validate present results.

On the other hand, this study has also certain advantages. All patients were recruited and followed in one regional center and thus represent homogeneous group of patients treated and surveyed in uniform way. Moreover, the present study was driven by previous observations on different patient group (also single center cohort) at the transcript level and thus congruent results obtained now cannot be attributed to the chance as in the case of hypothesis-generating screens.

Taken together, our results confirm the prognostic role of SLC22A3 protein in PDAC previously suggested by targeted transcriptomic study. ABCC2 transporter seems to play rather marginal role in this regard, but the effect of combinations of both biomarkers observed in a small scale subgroup analysis should be further validated by independent studies.

## Compliance with Ethical Standards

### Ethical approval

All procedures performed in studies involving human participants were in accordance with the ethical standards of the Ethical Commission of the Medical Faculty and Teaching Hospital in Pilsen, Czech Republic (reference no. 301/2019) and with the 1964 Helsinki declaration and its later amendments or comparable ethical standards. This article does not contain any studies with animals performed by any of the authors.

### Informed consent

Informed consent was obtained from all individual participants included in the study.

## Data Availability

All data generated or analyzed during this study are included in this published article.
